# Cell-Type Specific Expression of a Dominant Negative PKA Mutation in Mice

**DOI:** 10.1371/journal.pone.0018772

**Published:** 2011-04-12

**Authors:** Brandon S. Willis, Colleen M. Niswender, Thomas Su, Paul S. Amieux, G. Stanley McKnight

**Affiliations:** Department of Pharmacology, University of Washington, Seattle, Washington, United States of America; Medical College of Georgia, United States of America

## Abstract

We employed the Cre recombinase/*lox*P system to create a mouse line in which PKA activity can be inhibited in any cell-type that expresses Cre recombinase. The mouse line carries a mutant *Prkar1a* allele encoding a glycine to aspartate substitution at position 324 in the carboxy-terminal cAMP-binding domain (site B). This mutation produces a dominant negative RIα regulatory subunit (RIαB) and leads to inhibition of PKA activity. Insertion of a *lox*P-flanked neomycin cassette in the intron preceding the site B mutation prevents expression of the mutant RIαB allele until Cre-mediated excision of the cassette occurs. Embryonic stem cells expressing RIαB demonstrated a reduction in PKA activity and inhibition of cAMP-responsive gene expression. Mice expressing RIαB in hepatocytes exhibited reduced PKA activity, normal fasting induced gene expression, and enhanced glucose disposal. Activation of the RIαB allele *in vivo* provides a novel system for the analysis of PKA function in physiology.

## Introduction

The cyclic AMP-dependent protein kinase (PKA) holoenzyme exists as an inactive heterotetrameric complex of two catalytic (C) subunits that are bound and inhibited by two dimerized regulatory (R) subunits [1]. Cooperative binding of two cAMP molecules to each R subunit causes the release of active C subunit [2] and leads to downstream cellular changes in the activity of transcription factors, enzymes, ion channels, and many other cellular substrates [3], [4]. The mouse genome encodes four R subunit genes (RIα, RIβ, RIIα, RIIβ) and two C subunit genes (Cα and Cβ) [5]. Furthermore, both Cα and Cβ genes have alternative splice variants, thus adding to the diversity of PKA signaling pathways [6], [7]. Most tissues constitutively express the α subunits whereas the expression patterns of the β subunits are more restricted. A major obstacle in studies to delineate the role of PKA in specific physiological pathways has been our inability to obtain cell-type specific inhibition or activation of the kinase *in vivo*.

One approach to study the physiological role of PKA *in vivo* has relied on molecular genetic techniques to disrupt specific PKA subunit genes or overexpress mutant forms of PKA subunits. Each PKA subunit gene has been individually disrupted in mice [8]–[14] and despite the widespread expression patterns of R and C isoforms, only the disruption of RIα results in embryonic lethality [11]. Furthermore, in RIβ-, RIIα-, and RIIβ-null mice, the RIα subunit has the ability to compensate for the loss of other R subunits in several tissues and it has been suggested that RIα serves as a physiological “buffer” to limit the activity of free C subunit when it exists in excess of R subunit [15].

The R subunits have two cAMP binding sites (A and B) in the carboxyl terminal domain of the protein and mutations that disrupt cAMP binding to either site inhibit the ability of cAMP to activate the mutant holoenzyme [16]. In the present study, we used gene targeting to introduce a point mutation into exon 11 of the endogenous RIα allele. This point mutation results in a Gly to Asp substitution at amino acid 324 within the site B cAMP-binding domain in RIα and is identical to the site B mutation first characterized in S49 cells. This mutation prevents cAMP from binding to site B and produces a dominant negative phenotype in cell culture [17]–[20]. The introduction of a *lox*P-flanked neomycin resistance (neo^r^) cassette and polyadenylation signal into the intron upstream of the site B mutation prevents expression of the mutant RIα allele; however, in the presence of Cre recombinase, excision of the *floxed* neo^r^ cassette permits expression of mutant RIα. We use this strategy to activate the dominant negative allele in embryonic stem (ES) cells in culture and in hepatocytes *in vivo* and report the effects on kinase activation, gene regulation, and metabolic parameters.

## Results

### Analysis of the RIαB/Cre mutant phenotype in ES cells

**Figure 1:**
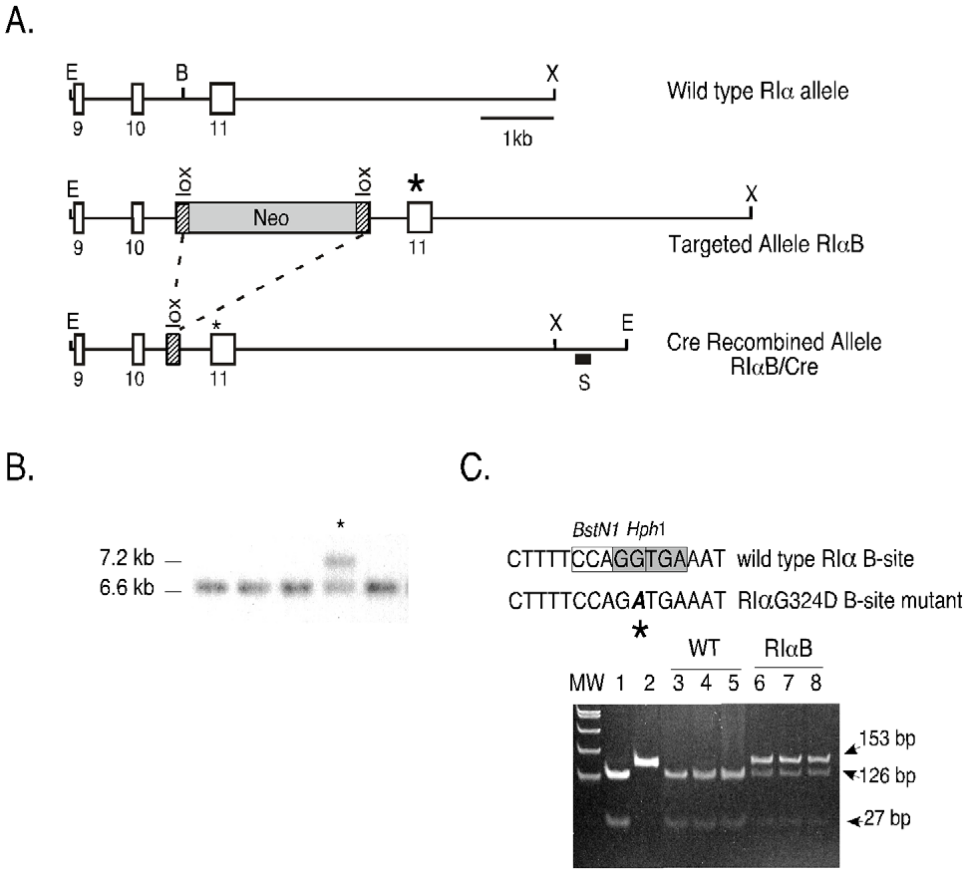
Targeting construct to generate RIαB mice. *A*, The *upper diagram* shows a simplified restriction map of a 6.6 kb fragment of the RIα genomic sequence encoding exons 9, 10 and 11 that was subcloned into Bluescript KS^+^. The *middle diagram* is the targeted allele (RIαB**)**, which contains the B site mutation (G324D) in exon 11 (as indicated by an asterisk) and a *lox*P-neomycin resistance cassette inserted into a *Bgl*II site in intron 10. The *bottom diagram* is the Cre modified mutant RIα allele containing a single *lox*P site (RIαB/Cre). Restrictions sites: *Eco*RI, E; *Bgl*II, B; *Xho*I, X. *B*, A Southern blot of *EcoR*V-digested genomic ES cell DNA using a 3′ *Sph*I/*EcoR*I genomic probe (as indicated by the black box). This strategy recognizes 6.6 kb fragment corresponding to the WT allele and a 7.5 kb fragment corresponding to the mutant RIα allele, RIαB. An asterisk indicates the correctly targeted clone. *C*, Identification of the B site mutation (as indicated by the asterisk) was confirmed by an *Hph*1-mediated restriction digest of a 153 bp PCR fragment generated with a primer set that amplifies within the site B cAMP domain of a WT (lane 1) and site B mutant plasmid vector (lane 2). In WT cells, *Hph*1 digestion of the 153 bp PCR product yields two products (126 bp and 27 bp) (lanes 3, 4, 5), whereas *Hph*1 digestion of the 153 bp amplicon from RIαB ES cells, which carry a single mutant allele and a single WT allele, results in three fragments: 153 bp (mutant), 126 bp (WT) and 27 bp (WT).

A 6.6 kb 129svJ mouse RIα genomic fragment containing exons 9–11 was used to generate the RIαB targeting construct. Site-directed mutagenesis was performed to introduce a single point mutation into exon 11 encoding the B site cAMP-binding domain. This mutation has been previously described to inhibit cAMP binding to the B site in the RIα subunit, thereby generating a PKA holoenzyme that is strongly resistant to cAMP activation [20]. The insertion of a *loxP*-flanked neo^r^ cassette into the intron upstream of the B site mutation effectively shuts off expression from the mutant allele, termed RIαB (Figure. 1A). REK2 mouse embryonic stem (ES) cells [8] (3 out of 324 analyzed) that had properly integrated the RIαB construct by homologous recombination were identified by Southern blot with a 3′ probe that distinguishes the wild type (WT) and floxed-neo^r^ mutant alleles (Figure 1B). The presence of the B site mutation was confirmed by polymerase chain reaction (PCR) and an *Hph1* DNA restriction digest (Figure 1C).

**Figure 2:**
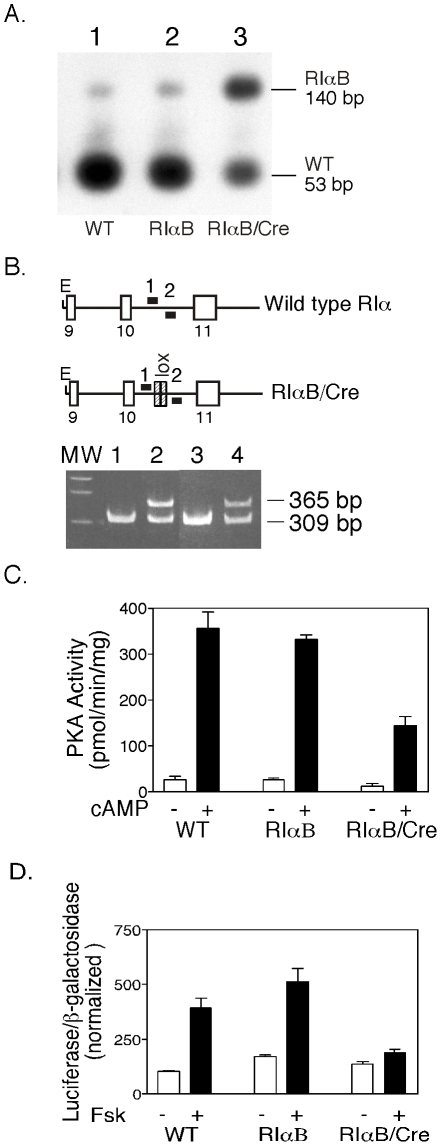
Cre recombinase*-*mediated activation of the RIαB allele in ES cells decreases PKA activity and forskolin-stimulated CRE-luciferase reporter expression. *A*, Transfection of ES cells with a *Cre* expression vector (pOG231) results in the expression of mutant RNA transcripts. Levels of mutant transcripts were determined by RT-PCR, restriction digestion, and Southern analysis as described in Materials and Methods. The 53 bp fragment indicates WT and the 140 bp fragment indicates mutant RIα transcripts. The small upper band present in lane 1 (WT) is due to incomplete digestion with BstN1, and was subtracted from lane 2 (RIαB) to determine the level of mutant RNA in these cells (<2%). The level of mutant transcript in RIαB/Cre ES cells is ∼49%. *B*, Detection of Cre-mediated recombination events in RIαB/Cre ES cells was determined by PCR using primers 1 and 2 (as shown in the figure) to amplify across the specified regions. Lane 1 is a WT control, Lane 2 is positive control for recombination. Lane 3 represents RIαB ES cells, which contain a single band due to presence of single WT RIα allele (the PCR conditions did not allow amplification through the floxed-neo^r^ cassette). Lane 4 represents RIαB/Cre ES cells, which contain a recombined allele (356 bp) and WT allele (309 bp). *C*, Kinase assay of basal and total activity in WT, RIαB, and RIαB/Cre ES cells. All samples were done in triplicate in the absence (basal PKA activity) or presence of 5 µΜ cAMP (total PKA activity) using Kemptide as substrate. Kinase activity that was not PKA-specific was measured in the presence of PKI and subtracted from basal and total values. Data values are represented as mean ± SEM. *D,* Representative CRE-luciferase assay. WT, RIαB, and RIαB/Cre ES cell lines were transfected with the CRE-dependent α168-luciferase reporter and then stimulated with forskolin (10 uM) for 14 h before an assay for luciferase activity as described in Materials and Methods. Transfections were done in triplicate.

To confirm the ability of the floxed-neo^r^ cassette to effectively disrupt expression of the mutant RIα allele, RT-PCR analysis of mRNA isolated from WT and RIαB ES clones was performed (Figure 2A). Amplification of the WT RIα gene generates an RT-PCR product of 140 bp that can be digested with either BstN1 (as shown in Figure 2A) or Hph1 to give a 53 bp fragment that is detected by Southern blot hybridization as described in Materials and Methods. We detected less than 2% mutant mRNA (the 140 bp fragment that is resistant to BstN1) in correctly targeted ES cell recombinants, indicating that the presence of the floxed-neo^r^ cassette with tandem poly A addition signals effectively precludes mRNA production from the mutant allele. ES cells containing this “silent” mutant allele are thus heterozygous at the RIα locus.

To determine whether the loss of a single RIα allele affected PKA activity, we performed kinase assays on RIαB and WT ES cells. No measurable changes were observed in kinase activity from ES cells harboring the RIαB allele compared to WT ES cells, suggesting that a single allele is sufficient to maintain wild type levels of PKA activity in ES cells (Figure 2C, RIαB lane). To determine if Cre-mediated excision of the floxed-neo^r^ cassette would result in expression of the mutant RIα subunit, we transfected a plasmid expression vector encoding Cre recombinase (Cre) into the ES cells. After recombination, a single 56 base pair fragment containing the 34 base pair *lox*P site and flanking nucleotides should remain in the intron and this small insertion was exploited to distinguish between WT (309 bp) and mutant (365 bp) alleles. The transfection strategy resulted in efficient removal of the floxed-neo^r^ cassette in approximately 15% of the RIαB ES cell clones, generating a transcriptionally active mutant RIα allele designated RIαB/Cre (Figure 2B). Since there is still one WT allele present in RIαB/Cre ES cell clones, the expected amount of total mutant mRNA should be 50%. We detected approximately 49% mutant mRNA in ES cells expressing the activated mutant RIα allele (Figure 2A, lane 3) indicating that expression of mRNA from both the WT and mutant allele was the same. The expression of the mutant protein in RIαB/Cre ES cells resulted in a 60% reduction in both basal and cAMP-stimulated kinase activity compared to unrecombined RIαB or WT ES cells (Figure 2C). However, the lower PKA activity associated with the expression of mutant RIα did not impair proliferation or survival of these ES cell recombinants (data not shown).

### Inducible CREB-dependent gene expression is attenuated in RIαB expressing ES cells

Several studies have shown that the C subunit can translocate to the nucleus and phosphorylate serine 133 on CREB, the transcriptional activator of cAMP-responsive genes [21], [22]. To determine if reductions in PKA activity caused by expression of RIαB could decrease CREB-dependent gene transcription, we transfected a cAMP-Responsive Element (CRE)-luciferase reporter construct into WT, RIαB, and RIαB/Cre ES cells. Basal transcription of the CRE-luciferase reporter was similar in all ES cells regardless of the state of the RIα alleles (Figure 2D). Treatment of both WT and RIαB ES cells with the adenylyl cyclase activator forskolin resulted in a three-fold induction in CRE-luciferase activity (Figure 2D). In contrast, forskolin-induced transcription was diminished by 90% in RIαB/Cre ES cells as shown in (Figure 2D, last lane). The capacity of RIαB protein (expressed from a single endogenous allele) to attenuate cAMP-inducible gene transcription in ES cells supports its application *in vivo* as a dominant inhibitor of PKA.

### RIαB mice have wild type levels of PKA activity

**Figure 3:**
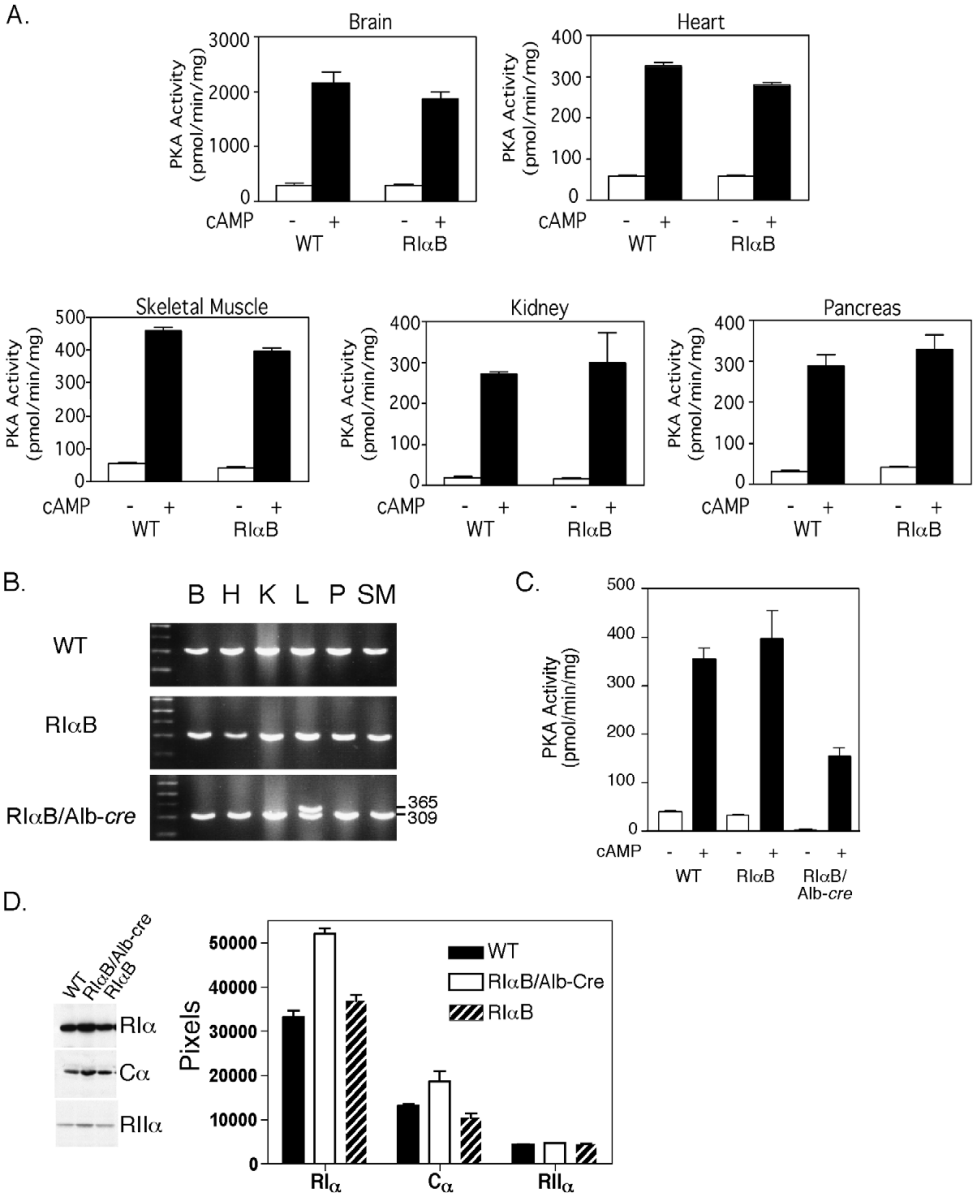
Cre-mediated activation of RIαB allele specifically in the liver decreases PKA activity in vivo. *A*, Kinase assay on tissue homogenates from WT and RIαB mice measured in the absence (basal) or presence (total) of 5 µΜ cAMP using kemptide as the substrate. Non-PKA specific activity was measured in the presence of PKI and subtracted from basal and total values. Data values are represented as mean ± SEM; n = 3 animals per group. *B*, Representative PCR analysis of genomic DNA of brain (B), heart (H), kidney (K), liver (L) pancreas (P) and skeletal muscle (SM) were examined for Cre*-*mediated recombination of the RIαB allele in WT, RIαB and RIαB/Alb-*cre* mice. A 309 bp fragment indicates WT RIα, whereas a 365 bp fragment indicates the recombined mutant RIα allele, which was restricted to the liver. *C*, Kinase assay on liver homogenates from WT, RIαB, and RIαB/Alb-*cre* measured in the absence (basal) or presence (total) of 5 µΜ cAMP using Kemptide as the substrate. Non-PKA specific activity was measured in the presence of PKI and subtracted from basal and total values. Data values are represented as mean ± SEM; n = 3 animals per group. *D*, Representative western analysis and quantitation of pixel density on film exposures from WT, RIαB/Alb-Cre and RIαB mouse liver extracts. Data values in bar graph are represented as mean ± SEM. n = 4 replicates.

The microinjection of correctly targeted RIαB ES cells into C57BL/6 blastocysts produced male chimeras that were subsequently bred to C57BL/6 females to produce F1 animals harboring a single RIαB allele and a wild type RIα allele. On a mixed 129Sv/J and C57BL/6 background, RIαB mice are healthy, fertile, and physically indistinguishable from WT littermates. However, on a higher percentage C57BL/6 background, RIαB male mice are subfertile. This result is consistent with our previous study of RIα heterozygous mice on a high C57BL/6 background [23]. Because insertion of the floxed-neo^r^ upstream of the mutated exon 11 is functionally equivalent to the targeted disruption of the RIα gene, intercrossing RIαB mice to produce homozygotes is predicted to result in early embryonic lethality similar the original RIα-null animals [11]. As predicted, no viable double mutant RIαB mice were born from the interbreeding of RIαB heterozygous mice. To determine if RIαB mice have normal levels of PKA activity in select tissues, kinase assays were performed on a panel of tissues including heart, kidney, brain, skeletal muscle, and pancreas. Tissues from RIαB mice expressed PKA activity comparable to WT mice, suggesting that one active RIα allele is sufficient to maintain PKA activity in most situations (Figure 3A). However, these RIαB animals have the same phenotypes that we have documented for RIα heterozygotes; this includes male infertility when the animals are on a C57BL/6 background and increased tumor susceptibility as the animals age [23], [24].

### Liver-specific expression of the mutant R subunit reduces PKA activity

To test whether the inhibitory mutant RIα allele could be activated *in vivo*, we mated RIαB mice to transgenic mice expressing Cre recombinase under the control of the albumin promoter (Alb*-cre*). This promoter has been demonstrated to restrict expression of *Cre* recombinase to liver hepatocytes [25]. Previous studies have shown that Alb*-cre* transgenic animals obtain maximum recombination levels of floxed alleles at 5 to 8 weeks of age [26]. From birth through adulthood, RIαB/Alb*-cre* animals appear to be healthy and physically indistinguishable from aged-matched littermates. Genomic DNA analysis of brain, heart, kidney, liver, pancreas and skeletal muscle from 8-week old male mice revealed Cre-mediated recombination only in liver DNA of RIαB/Alb-*cre* mice (Figure 3B). Kinase assays revealed that the presence of the mutant RIα subunit in liver extracts caused a significant reduction in both basal and cAMP-stimulated PKA activities compared to WT and RIαB mice (Figure 3C). Since the estimated percentage of cells expressing albumin in liver is close to 60% [27], it follows that the actual inhibition of PKA in hepatocytes may be greater than we observe for whole liver. These results demonstrate that the expression of a single mutant RIα allele is sufficient to produce a dominant inhibition of PKA activity *in vivo*. Western analysis of liver extracts from WT, RIαB and RIαB/Alb-Cre mice revealed an increase in RIα and Cα protein levels in the RIαB/Alb-Cre mice; in contrast, RIIα protein levels were similar in all three genotypes (Figure 3D).

### Effects of RIαB activation on liver gene expression

**Figure 4:**
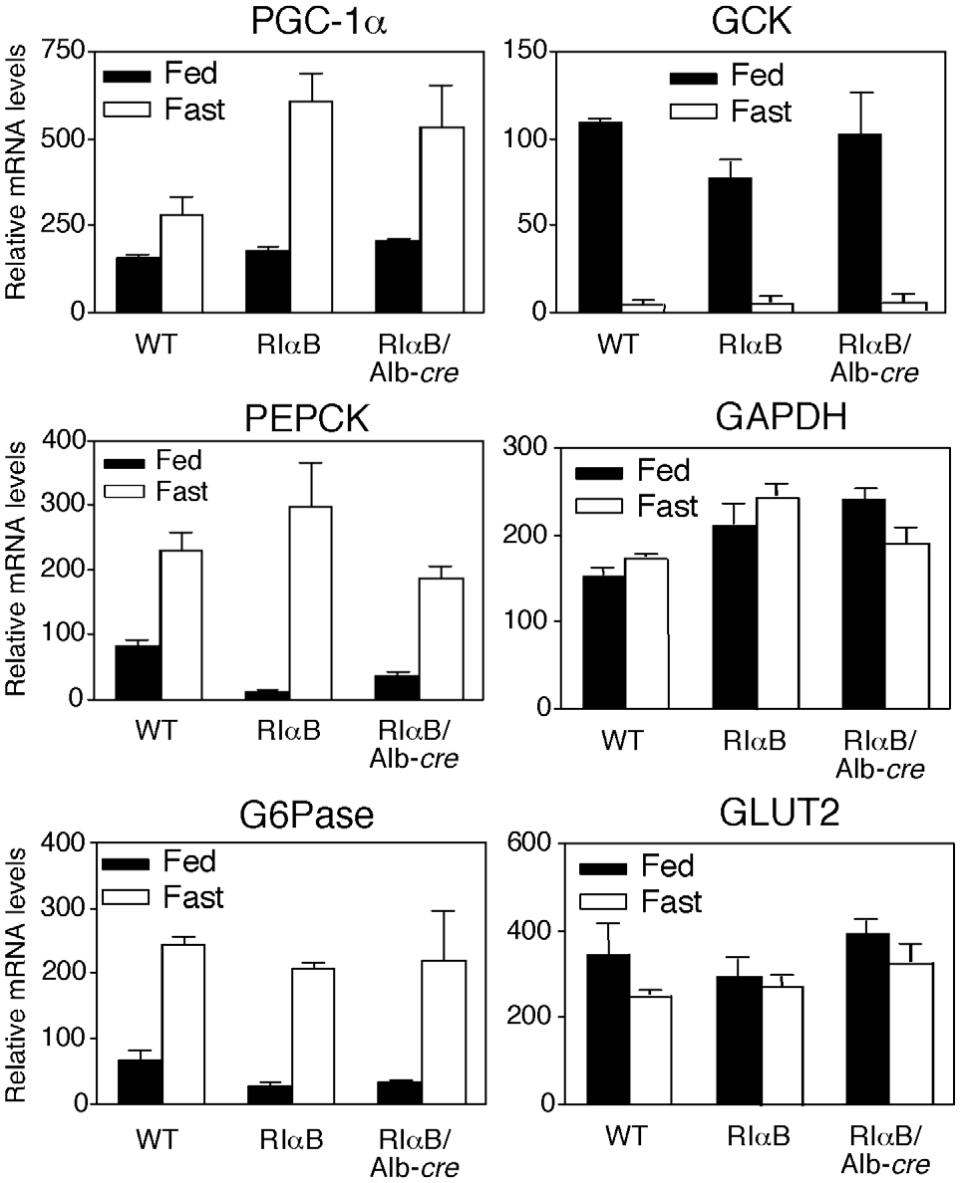
Liver-specific PKA inhibition does not alter fasting-regulated gene expression. mRNA levels of enzymes required for gluconeogenesis in the 24 hr fasted state (Fast) or after being fasted for 24 hr then allowed access to food for 6 hr (Fed) in WT, RIαB**,** and RIαB/Alb-*cre* mice. Levels of each mRNA were measured by quantitative real time PCR as described in Materials and Methods. Data are expressed relative to the results of a fasted WT control animal. Each value represents the mean ± SEM; n = 3 animals per group. No significant differences were found in gene induction. PGC-1α (peroxisome proliferator-activated receptor-γ coactivator 1α), PEPCK (phospho*enol*pyruvate carboxykinase), G6Pase (glucose-6-phosphatase), GCK (glucokinase), GAPDH (glyceraldehyde 3-phosphate dehydrogenase), and GLUT2 (glucose transporter type 2).

Because expression of the RIαB protein significantly inhibited cAMP-mediated induction of a CRE-luciferase reporter construct in ES cells, we investigated whether inhibition of liver PKA *in vivo* would specifically block the induction of potential CREB-dependent genes important for liver function. Two CRE-containing genes, phospho*enol*pyruvate carboxykinase (PEPCK) and glucose-6-phosphatase (G6Pase), both of which are induced in response to fasting, were investigated [28], [29]. It has also been reported that PKA-mediated phosphorylation of CREB stimulates the expression of PGC-1α (peroxisome proliferator-activated receptor-γ coactivator 1α) and this transcriptional coactivator strongly induces the expression of several gluconeogenic enzymes, including PEPCK and G6Pase [30], [31]. Surprisingly, liver-specific reductions in PKA had no significant effect on the induction of PGC-1α, PEPCK or G6Pase during fasting conditions, as shown in Figure 4. Following a 6 hr refeeding period, PEPCK, G6Pase and PGC-1α transcripts all returned to basal levels despite the expression of RIαB. As a control the levels of Glut2 and GAPDH mRNA are shown and they were not significantly changed by either nutritional state or genotype. The mRNA levels for glucokinase (GCK) are inhibited during fasting by a cAMP-dependent mechanism and induced by insulin in fed animals [32]. However, GCK also responded normally in both RIαB and RIαB/Alb-cre mice compared with WT (Figure 4). Collectively, these data show that despite liver-specific reductions in PKA activity, the induction of genes essential for gluconeogenesis (PGC1α, PEPCK, and G6Pase) and the repression of GCK remain functionally intact.

### Whole body parameters are normal in RIαB/Alb-cre mice

Between 6 to 12 weeks of age, no significant differences were found in body weight and or liver weight of RIαB/Alb-*cre* mice compared to RIαB or WT mice (Table 1). To investigate the effects of a PKA deficiency in the regulation of blood glucose homeostasis, individual mice were subjected to fasting and refeeding conditions, and then assayed for plasma blood glucose and insulin levels. All three genotypes had similar decreases in plasma blood glucose and insulin after a 24 hr fasting period (Table 1). After a 24 hr fast, refeeding for six hours produced similar increases in plasma blood glucose and insulin. No differences were observed between genotypes with respect to plasma non-esterified fatty acids (NEFA) or triglycerides during fasting or feeding conditions (Table 1).

**Table 1:** Plasma glucose, insulin, and metabolite concentrations in 24 hr fasted or 24 hr fasted and 6 hr refed animals.

Genotype	Body weight(g)	Liver wt (% body wt)	Blood Glucose (mg/dl)		Insulin (ng/ml)		Non-esterified fatty acids (mEq/L)		Triglycerides (mg/dl)	
			Fast	Refed	Fast	Refed	Fast	Refed	Fast	Refed
WT	27.3±.8	4.5±.1	73±3	103±3	.63±.04	2.0±.3	2.2±.1	.38±.02	57±4	28±2
RIαB	27.3±.8	4.8±.4	72±3	109±3	.56±.07	1.8±.3	2.4±.1	.44±.03	55±6	29±3
RIαB/Alb-cre	27.7±.7	4.8±.2	66±4	105±2	.76±.15	1.68±.5	2.2±.2	.47±.04	56±4	28±2

Age-matched male mice 12 to 18 weeks old were bled after a 24 hr fast and 6 hr refeeding. Blood for all measurements was obtained from the hindlimb saphenous vein. Each value represents the mean ± SEM. No significant differences were found in any of these parameters. For liver weight determination, n = 5 animals per genotype and for all other measurements, n = 7–16 animals per genotype.

### Enhanced glucose disposal in RIαB/Alb-cre mice

**Figure 5:**
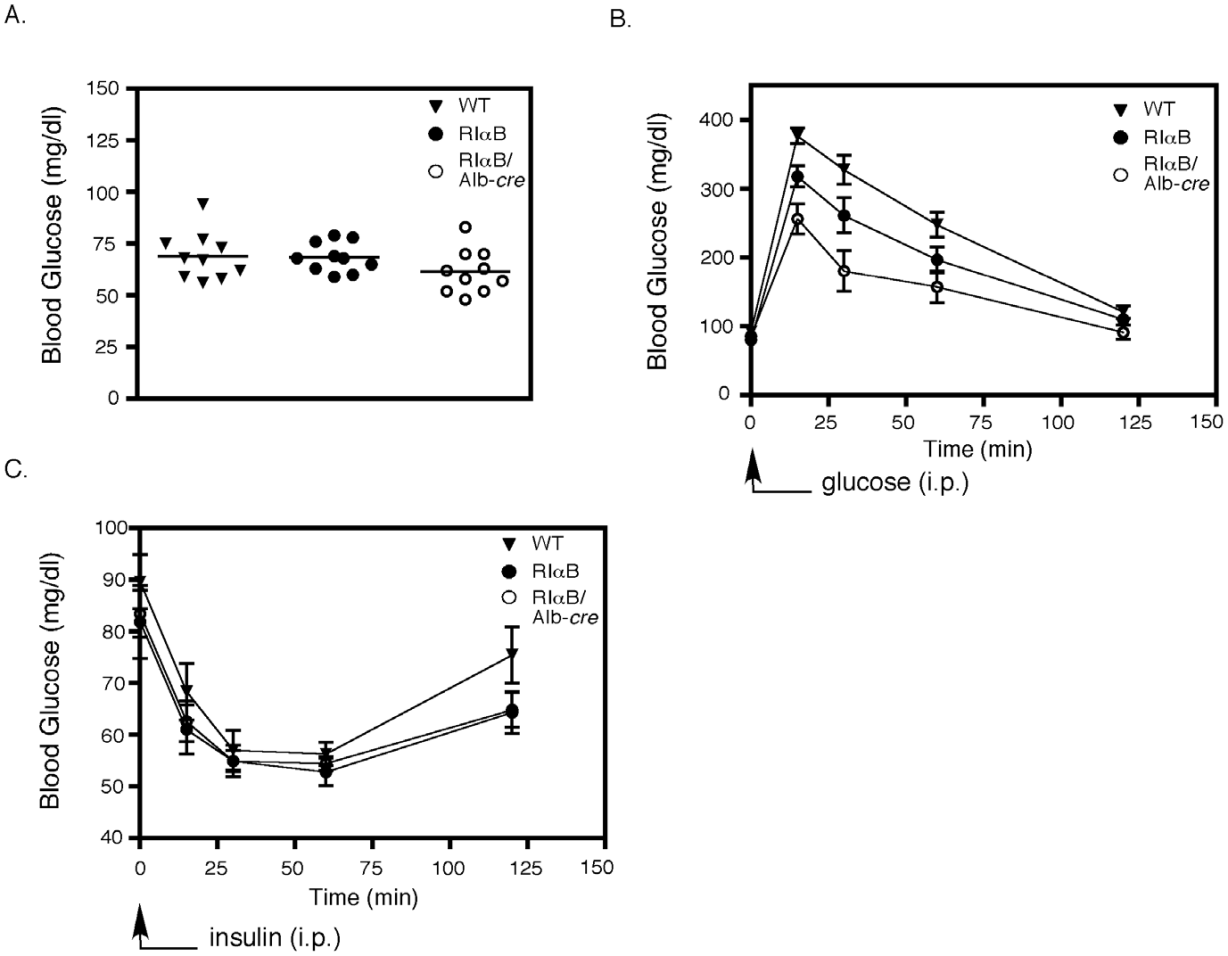
RIαB/Alb-*cre* mice exhibit increases in glucose disposal. *A*, Blood glucose levels were determined in 24 hr fasted WT, RIαB, and RIαB/Alb-*cre* mice. n = 10. *B*, Glucose tolerance test was performed in 24 hr fasted WT, RIαB, and RIαB/Alb-*cre* mice injected i.p. with 2 mg of glucose per gram of body weight. Blood glucose levels were determined at the indicated time points. Over the first 60 min, the glucose disposal of RIαB/Alb-cre was significantly enhanced compared to RIαB alone (p<.001). n = 7. *C*, WT, RIαB, and RIαB/Alb-*cre* mice were injected with 0.75 mU of insulin per kilogram of body weight at time 0 and blood glucose levels were determined at the indicated time points. Values are presented as means ± SEM. n = 10.

Despite the apparently normal physiology of the RIαB/Alb-cre mice during fasting and refeeding, we hypothesized that they might respond differently when directly challenged by an infusion of glucose. RIαB/Alb-*cre* mice were fasted for 24 hr and then injected i.p. with 2 mg of glucose per gram of body weight and blood glucose levels were measured at the indicated time points. Basal glucose levels measured after the 24 hr fast and before the glucose challenge did not reveal a difference between WT, RIαB, and RIαB/Alb-Cre mice, although the RIαB/Alb-Cre mice did show a trend of lower glucose levels that did not reach statistical significance (Figure 5A). However, with the glucose challenge, RIαB/Alb-*cre* mice exhibited a significant improvement in total body glucose disposal compared to both WT and RIαB mice at 15, 30, and 60 min time points (Figure 5B). Although RIαB/Alb-cre mice demonstrated enhanced glucose disposal, their acute response to an insulin challenge appeared normal (Figure 5C).

### Removal of the other major regulatory subunit in liver from RIαB/Alb-cre mice does not alter the enhanced glucose disposal

**Figure 6:**
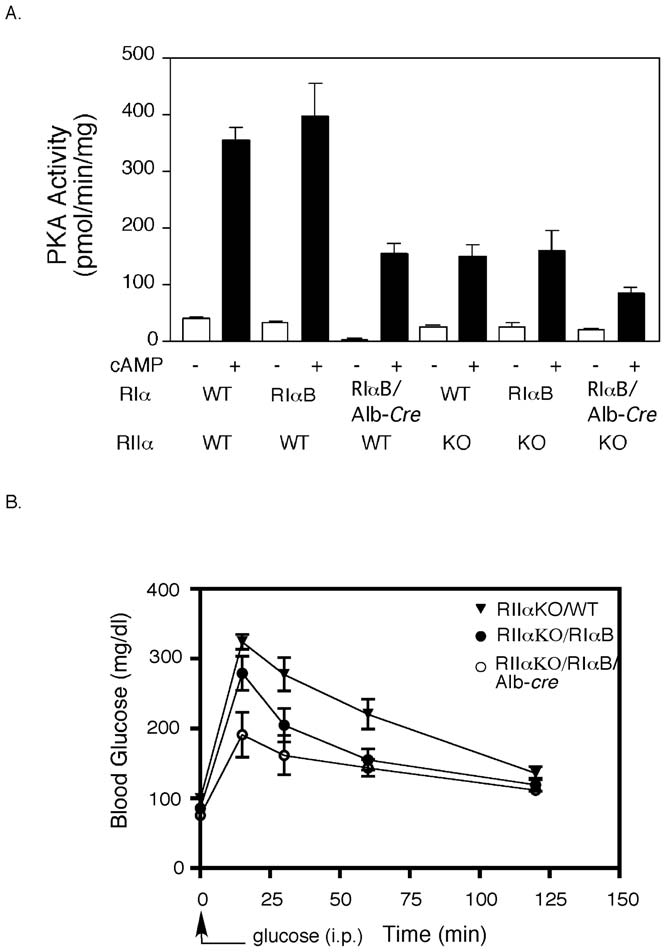
RIαB/Alb-*cre* mice and RIαB mice on the RIIα KO background exhibit increases in glucose disposal. *A*, Representative kinase assay of basal and total kinase activity in WT, RIαB, RIαB/Alb-Cre,RIIαKO, RIαB:RIIαKO, and RIαB/Alb-Cre:RIIαKO liver. All samples were done in triplicate in the absence (basal) or presence (total) of 5 µΜ cAMP using Kemptide as substrate. Kinase activity that was not PKA-specific was measured in the presence of PKI and subtracted from basal and total values. Data values are represented as mean ± SEM. At least three mice from each genotype were used for the kinase assay. *B*, Glucose tolerance tests on 24 hr fasted WT, RIαB and RIαB/Alb-Cre mice all crossed onto the RIIα KO background are shown. Two mg of glucose per gram of body weight was injected i.p. and blood glucose levels were determined at the indicated time points. Values are presented as means ± SEM. n = 5. Glucose levels in RIαB/Alb-cre animals were significantly reduced compared to WT and RIαB during the first 30 min. P<0.01.

Liver expresses both RIα and RIIα and the mutant RIαB protein expressed after Alb-cre induced recombination must compete for C subunits with both WT RIα and RIIα. In Figure 3 we demonstrated that expression of the RIαB allele was only able to inhibit PKA activity by about 60%. We reasoned that genetic deletion of the other liver PKA regulatory subunit (RIIα) on the RIαB/Alb-cre background might allow us to achieve greater inhibition of PKA activity. As shown in Figure 6A, kinase activity in whole liver homogenates was reduced by about 80% in RIIα KO:RIαB/Alb-cre mice and some fraction of the remaining 20% of PKA activity is likely to come from the non-hepatocyte cell types of the liver. Glucose tolerance tests on the RIIα KO:RIαB/Alb-cre mice demonstrated a greatly enhanced glucose disposal, exceeding that observed in RIαB/Alb-cre mice on a WT background (Figure 6B). A trend toward enhanced glucose disposal was also observed in RIαB mice in the absence of Cre recombinase (Figure 5B, 6B), suggesting the mice heterozygous for RIα exhibit improved glucose disposal.

### RIαB as a tool to study PKA-specific roles in virtually any cell type

Our results demonstrate the ability of the floxed RIαB mouse model to effectively inhibit PKA activity *in vivo* in specific cell types. We have demonstrated the utility of this strategy in collaboration with other laboratories to determine the role of PKA in development of the enteric nervous system [33] and in class-switch recombination in the immune system [34]. Additionally, we have crossed the RIαB mouse to several Cre Driver mouse lines and have observed significant alterations in the physiology of target tissues (Table 2). The ability to inhibit PKA activity *in vivo* in a cell-type-specific manner represents a significant addition to the technologies available for asking questions about the role of PKA in different biological processes.

**Table 2:** Phenotypes Observed by Activating the RIαB Allele with Various Cre Driver Mouse Lines.

Cre Driver	Tissue/cell types undergoing recombination	Observed Phenotype	Reference
Albumin-*Cre*	liver hepatocytes	enhanced glucose disposal with glucose challenge	this manuscript
AP2-*Cre*	BAT, partial heart and WAT	edema-related complications	B.S. Willis and G.S. McKnight, unpublished observations
Cd21-*Cre*	Mature B cells	impaired class-switch recombination (CSR)	Vuong et al.(2009)
Darpp32-*Cre*	striatum	short limbs; lean; hypophagic; impaired rotarod performance	L.Yang and G.S. McKnight, unpublished observations
Hox11L1-*Cre*	neural crest-derived enteric neurons	lethal intestinal pseudo-obstruction	Howe et al.(2006)
PLP-*Cre*	neural crest-derived tissues, CNS, PNS and ENS	lethal intestinal pseudo-obstruction	Howe et al.(2006)
ROSA26-*Cre*	ubiquitous	embryonic lethality	B.S. Willis and G.S. McKnight, unpublished observations
Sf1-*Cre*	VMH hypothalamus; testis; ovary; pituitary; adrenal	females appear normal; males appear normal with smaller testis and seminal vesicles	L. Yang and G.S. McKnight, unpublished observations
Sim1-*Cre*	PVN hypothalamus; kidney collecting duct	nephrogenic diabetes insipidus	M. Gilbert, L. Yang and G.S. McKnight, unpublished observations

## Discussion

We have utilized the Cre*/lox*P recombination system to devise a novel strategy to produce lesions in the PKA signaling pathway *in vivo*. The ES cells and mice that we have created carry a silenced RIα mutant allele (RIαB) that, when activated by Cre recombinase, expresses an RIα protein that is defective in cAMP binding. Holoenzymes composed of this mutant RIα subunit require nearly 100 fold higher concentrations of cAMP for half maximal activation compared to native holoenzymes [16], [20] We have activated this silenced allele in ES cells and demonstrate that expression of the RIαB protein affects PKA-dependent kinase activity as well as cAMP-induced gene expression. Activation of the mutant RIα allele in hepatocytes using *Alb-cre* transgenic mice results in a substantial decrease in PKA activity only in the liver, demonstrating that the RIαB mice can be used to elicit a dominant negative PKA effect in a cell-specific manner in vivo. Although kinase activity is clearly reduced when the RIαB allele is activated, we do observe an increase in RIα and Cα protein levels, suggesting that PKAI holoenzyme is stabilized in the inactive state; this is consistent with previous work from our laboratory demonstrating that RIα and Cα proteins are more stable when incorporated into holoenzyme [15] and also with our unpublished data using other Cre Driver mouse lines crossed to the RIαB mouse (Table 2).

One important criterion that must be established before using the Cre/*lox*P system in mice is that the RIαB allele is effectively silenced prior to Cre recombination. We have demonstrated that insertion of a *floxed*-neo^r^ gene into intron 10 upstream of the site B mutation in exon 11 conditionally regulates expression of the mutant RIα allele. As demonstrated by RT-PCR experiments, when the *floxed*-neo^r^ cassette is present, the silent RIαB allele effectively renders ES cells and mice heterozygous at the RIα locus. The *floxed*-neo^r^ gene employed in this study contains splice donor/acceptor sites from the SV40 small T antigen and tandem poly A addition signals and is inserted in the same orientation as the gene. It is likely that the poly A addition sequence causes early termination of the mutant transcript.

Once activated by Cre-mediated excision of the *floxed*-neo^r^ cassette, ES cells and hepatocytes expressing the RIαB protein exhibit significant reductions in basal and cAMP-stimulated PKA activity. However, this approach does not completely eliminate PKA activity as can be seen for RIαB expressing ES cells where all of the cells have activated the RIαB allele and yet 40% of the PKA activity remains. We have previously shown that overexpression of a dominant negative RIα protein, R(AB), in cell culture completely inhibits PKA activity [19]. This difference may be due in part to the fact that the dominant-negative R(AB) subunit is completely resistant to cAMP activation whereas the single RIαB site mutation (G324D) created in our mice can still respond to high levels of cAMP (K_a_ = 4.7 µM cAMP)[20]. Probably a more significant difference is that stable R(AB) transformants in cell culture contain many copies of the R(AB) construct driven by robust promoters that produce a large excess of mutant protein. In our ES and mouse model systems, a weaker mutant (RIαB) is being transcribed from only a single allele under the control of the endogenous RIα promoter and this may not be adequate to sequester all of the available C subunits.

The cAMP-dependent regulation of gene expression is thought to occur in part through the phosphorylation of CREB/ATF transcription factors bound to DNA regulatory sequences known as CREs located in the promoter of many genes [35]. Transcriptional activation is initiated by phosphorylation of serine 133 in CREB by the C subunit of PKA and studies have shown that translocation of C into the nucleus stimulates transcription of CRE-containing genes [21], [36], [37]. It has been demonstrated that expression of R(AB) in various cell lines blocks cAMP-responsive gene induction of CRE-containing promoters [22], [38]. In this paper we show that attenuation of PKA activity by RIαB activation in ES cells also blocked the ability of cAMP to induce transcription from a CRE-luciferase reporter construct.

The ability of RIαB to inhibit CREB-dependent gene expression in ES cells prompted us to examine the regulation of genes within the gluconeogenic pathway in the liver of RIαB expressing mice. Several of the genes essential for gluconeogenesis have been identified as CREB-regulated (including PEPCK and G6Pase) and are thought to be induced by PKA activation [29], [39]. CREB has also been shown to induce the transcriptional coactivator PGC-1α in the liver [40], which regulates the expression of several gluconeogenic enzymes including PEPCK and G6Pase [30]. We were surprised to find that PGC-1α, PEPCK, G6Pase, and GCK were all expressed and regulated normally by nutritional state in livers expressing the dominant negative RIαB allele. One explanation for these results is that the PKA inhibition by RIαB is not complete and sufficient kinase activity remains to support gene regulation.

An alternative view, which we favor, is that the complex nutritional/hormonal regulation of these genes in a physiological setting facilitates compensation for the partial loss of PKA activity and tends to maintain the normal levels of gene expression. In addition to CREB itself, the gluconeogenic program is modulated by CREB-regulated transcription coactivator 2 (CRTC2), which is phosphorylated by Salt Inducible Kinase 2 (SIK2) and excluded from the nucleus by binding to 14-3-3 proteins. Fasting-dependent increases in glucagon result in a dephosphorylation of CRTC2, which then allows CRTC2 to enter the nucleus and promote transcription of CREB target genes by interacting with the b-ZIP domain of CREB [41]. Insulin inhibits CRTC2-dependent induction of gluconeogenesis via Akt-dependent phosphorylation and activation of SIK2, resulting in phosphorylation of CRTC2 and ubiquitin-dependent degradation of CRTC2 in the proteosome [42]. Insulin also potently inhibits gluconeogenesis through the activation of PI3 kinase, resulting in Akt phosphorylation of FOX01 and exclusion of FOX01 from the nucleus [43], [44]. The lack of an absolute requirement for PKA activity in gluconeogenic gene expression is also supported by the finding that disruption of the CREB-CBP interaction in liver does not attenuate expression of gluconeogenic genes such as PEPCK, G6Pase and PGC-1α [41].

The observation that RIαB/Alb-*cre* mice exhibit improved glucose disposal was unanticipated, since these animals properly adjust blood glucose and insulin levels during periods of fasting and refeeding. Based on our gene expression data, the improvement in glucose disposal in these mice cannot be directly linked to impairments in gluconeogenic gene expression. In the liver, there is a balance between insulin and cAMP signaling pathways and dysregulation of this equilibrium has been suggested to be one of the underlying mechanisms involved in the development of type 2 diabetes [45]. Perhaps the downregulation of PKA in the liver has shifted the balance between these two pathways, favoring insulin-like tone and improving the clearance of glucose from the blood stream. Further studies are required to elucidate the precise role of liver-specific PKA inhibition on whole body glucose disposal. Because of the many roles associated with PKA function in liver, it also remains to be determined if reductions in PKA activity alters other aspects of liver physiology such as regeneration [46] or xenobiotic transformation [47].

Our strategy renders mice heterozygous at the RIα locus, and expression from a single wild-type RIα allele appears to be adequate to maintain wild type PKA activity levels in most tissues. However, we have recently discovered that RIα heterozygosity alone produces phenotypes that include sperm abnormalities and subsequent infertility [23] as well as late onset development of soft tissue sarcomas, hemangiosarcomas, chondrosarcomas and hepatocellular carcinomas [24]. Experiments to understand the consequences of RIα heterozygosity on male fertility have been conducted in RIα heterozygous mice on a C57BL/6 genetic background. Isolation of mature sperm from these mice reveals profound morphological abnormalities with frequent broken heads and ruptured tails. This phenotype is reversed by mating RIα heterozygotes onto the Cα heterozygote background, demonstrating that sperm defects are due to unregulated C subunit activity at some point in development [23]. Interestingly, somatic mutations that result in a similar loss of function have been described in the human RIα locus *(PRKAR1α)* in patients with Carney complex, a familial multiple neoplasia syndrome characterized by cardiac and extracardiac myxomas, schwannomas, endocrine and gonadal tumors [48]–[50]. The possibility of these and other phenotypes associated with the unactivated RIαB mouse line needs to be considered and controlled for when interpreting physiological studies with these animals.

As a genetic tool, the RIαB mice can serve as a starting point to study the role of cAMP/PKA signaling *in vivo*. Using a very similar Cre/lox strategy we have previously reported the development of a mouse line that expresses a constitutively active Cα subunit (CαR) in response to activation by Cre recombinase [51]. Using these two mouse lines (RIαB and CαR), PKA activity can be modulated in either direction *in vivo* in any cell type for which a Cre Driver mouse exists. Evidence from crosses with other Cre-expressing mice has revealed the utility of RIαB in elucidating the effects of PKA in physiology. When RIαB is expressed specifically in neural crest progenitor cells using the PLP-*cre* and Hox11L1-*cre*, RIαB expression leads to craniofacial dysmorphism and severe enteric dysfunction [33]. Expression of RIαB in B cells disrupts the PKA dependent regulation of class switch recombination [34]. Expression of RIαB in the collecting duct cells of the kidney disrupts aquaporin-2 expression and function, causing diabetes insipidus (M. Gilbert, L. Yang, and G.S. McKnight, unpublished observations). Due to the broad expression pattern of RIα, this approach to selectively inhibit PKA activity using the Cre/*lox*P system should prove useful for the study of PKA signaling in many different cellular and physiological responses *in vivo.*

## Materials and Methods

### Ethics statement

All mouse procedures were approved under protocol 2022-01, titled “Regulation of cAMP-Dependent Protein Kinase Genes, by our Institutional Animal Care and Use Committee (IACUC) at the University of Washington, which operates under approval number A3464-01 from the Association for Assessment and Accreditation of Laboratory Animal Care (AAALAC).

### RIαB targeting vector

To generate the site B mutation (G324D) in the RIα protein, a 6.6 kb genomic *EcoR*I fragment containing exons 9, 10, and 11 of the RIα locus was isolated as previously described [11]. The genomic fragment was subcloned into a pUC18 vector and site-directed mutagenesis (QuikChange™ Stratagene) was performed to introduce a single G to A mutation in exon 11 using the oligonucleotide sequences 5′-TCTTTTCCAGATGAATTGCCCTGCTGA-3′and 5′-AGCCAGGGCAATTTCATCTGGAAAAGAGA-3′. A *lox*P flanked neomycin selection cassette containing the SV40 promoter, the neomycin phosphotransferase gene, and two SV40 polyadenylation sequences was inserted into a unique *BglII* restriction site between exons 10 and 11. 120 µg of the targeting vector was linearized at an unique *Not*1 site and electroporated into 1.2×10^7^ mouse REK2 ES cells [8] using a Progenitor™ II PG200 electroporator at 240 mV, 500 µF (Hoefer Scientific Instruments) and then selected in G418 (280 µg/ml) and 1000 U/ml leukemia inhibiting factor (LIF) for approximately 7 days before colonies were picked. DNA isolated from ES clones was digested with *EcoR*V and Southern blot analysis using Church and Gilbert hybridization buffer (250 mΜ sodium phosphate pH 7.2, 1 mΜ EDTA, 1% BSA, 7% SDS at 68°C) was performed to identify clones with the correct integration of the targeting vector using a 225 bp *Sph*I/*EcoR*I RIα genomic fragment located 3′ to the targeting vector. Identification of the site B mutation was confirmed by PCR analysis using the oligo sequences 5′-CGCTTGAGGATGTCTGAGCAC-3′and 5′-GTGATAGTCGTTACTGTCTC-3′ (94°C for 1 min followed by cycles of 94°C for 30 sec, 62°C for 30 sec and 72°C for 1 min for 29 cycles). Amplified PCR products were then digested with *Hph*1 (New England Biolabs) and analyzed on 14% acrylamide gels. The 153 bp DNA fragment corresponds to the site B mutant RIα allele and the 126 bp and 27 bp DNA fragments correspond to the WT RIα allele. Positively identified clones were microinjected into C57BL/6 blastocysts that were then implanted into pseudopregnant females. Male chimeric offspring were bred with C57BL/6 females and RIαB agouti offspring were identified by Southern blot and PCR analyses as described above.

### Transient transfection of ES cells with *Cre* recombinase

2×10^7^ RIαB ES clones were electroporated with 50 µg of pOG231, an expression vector encoding Cre recombinase driven by a CMV promoter (a generous gift from Stephen O'Gorman, Salk Institute, San Diego, CA) and seeded at ∼100 cells/cm^2^ on PMEF feeder layer in 10 cm dish. Individual ES colonies were picked and allowed to expand.

### Detection of Mutant RIα transcript

Expression of the mutant RIα mRNA was confirmed by Reverse Transcription-PCR analysis using the following oligonucleotides: 5′-CGAAAGAGGAAGATGTATGAA-3′ (exon 8) and 5′-GGACAGGGACACGAAGCTGTT-3′ (exon 11) (94°C for 1 min followed by cycles of 94°C for 30 sec, 62°C for 30 sec and 72°C for 1 min for 29 cycles) which amplifies a 140 bp fragment from both WT and mutant RIα cDNAs. PCR products were digested with BstN1, which overlaps the site of the point mutation and only cuts the WT product. The digested fragment was separated on a 2% agarose gel, and subjected to Southern blot analysis using a P^32^-end labeled oligonucleotide (5′-AGCCAGGGCAATTTCATCTGGAAAAGAGA-3′). The blot was exposed to X-Omat AR film for 3 hr and bands were quantified using NIH Image. The 53 bp band corresponds to WT RIα mRNA and the 140 bp band corresponds to RIαB mRNA.

### Recombination PCR analysis across *lox*P site

A PCR-based screening method using the oligonucleotide sequences 5′-TGATGGGATTGAATGTAGGC-3′ and 5′-TTAGAACAAGCTTCACTGTAC-3′ (94°C for 1 min followed by cycles of 94°C for 30 sec, 62°C for 30 sec and 72°C for 1 min for 29 cycles) was used to determine the presence of the single *lox*P site that remains after Cre-mediated recombination of the floxed neomycin resistance cassette within the RIαB allele. The 309 bp fragment corresponds to the WT RIα allele and the 365 bp fragment corresponds to the recombined mutant RIα allele.

### Transient transfection of ES cells with a CREB-dependent luciferase reporter

REK2 ES clones [8] were seeded at a density of 1.8×10^5^ cells/well in a 24 well plate before being transiently transfected using calcium phosphate precipitation with 2.5 ng of a CRE reporter gene (α168-luciferase), 50 ng of RSV β-galactosidase as an internal control for transfection efficiency and carrier plasmid pBluescript KS^+^ (Stratagene) to bring the total amount of DNA to 250 ng/well. Precipitates remained on the ES clones for 20 to 24 hr at 37°C in a 3% CO_2_ incubator. To induce expression of α168-luciferase, ES cells were incubated in DMEM supplemented with 2.5% fetal bovine serum ± 10 µΜ forskolin for 14 hr in 5% CO_2_. After forskolin treatment, ES cells were washed once in ice cold phosphate buffered saline, lysed, and harvested for reporter analysis as described previously [52]. Transfections were performed in triplicate and luciferase activity was normalized to RSV β-galactosidase activity for each sample.

### Generation of RIαB/Alb-*cre* mice

Hybrid (50% C57BL/6 and 50% 129SvJ) RIαB heterozygous mice were backcrossed 3 or more generations with C57BL/6 mice to produce RIαB mice on a >93% C57BL/6 genetic background. Albumin-*cre* (Alb-*cre*) transgenic mice were purchased from The Jackson Laboratory, Bar Harbor, ME. Because RIαB males on a high C57Bl/6 background are partially infertile, female RIαB mice were crossed to male Alb-*cre* transgenic mice to generate RIαB/Alb-*cre* mice. Only male mice were used in these experiments. All mice were maintained on a 12 hr light and 12 hr dark cycle with normal mouse chow and water in an SPF facility.

### Kinase assays

Protein extracts from ES cells, liver or tissues from *ad libitum* fed 8- to 10- week old male mice were prepared in ice-cold homogenization buffer (20 mΜ Tris, pH 7.5, 250 mΜ sucrose, 0.1 mΜ EDTA, 0.5 mΜ EGTA, 1% Triton X-100, 10 mΜ dithiothreitol, 1 µg/ml leupeptin, 3 µg/ml aprotinin, 40 µg/ml soybean trypsin inhibitor, 0.5 mΜ 4-(2-aminoethyl)benzenesulfonyl fluoride) using a dounce homogenizer followed by brief sonication (Branson Sonifier 250). Protein extracts were snap-frozen in liquid nitrogen and stored at −80°C. Protein extracts were thawed on ice and Bradford assays (BioRad) were performed to determine protein concentration of each extract. Kinase assays were performed in triplicate using the PKA substrate peptide Kemptide (Leu-Arg-Arg-Ala-Ser-Leu-Gly) in the presence or absence of 5 µΜ cAMP [20]. Non-specific PKA activity was measured in the presence of 4 µg/ml protein kinase inhibitor (PKI) peptide and subtracted.

### Western analysis

Western analysis, including description of the polyclonal antibodies to RIα, Cα, and RIIα, has been described [15]. Westerns were analyzed using the Image J software available by ftp at zippy.nimh.nih.gov/or http://rsb.info.nih.gov/nih-imageJ; developed by Wayne Rasband, National Institutes of Health, Bethesda, MD.

### Plasma blood glucose, insulin, and metabolite measurements

8 to 12 week old male mice were fasted for 24 hr or fasted for 24 hr followed by free access to chow for 6 hr. Blood from the from hind limb saphenous vein was collected and blood glucose levels were immediately measured using the One Touch II glucose meter (LifeScan, Milpitas, CA). Serum insulin levels were determined using a mouse insulin ELISA kit (Mercodia, Metuchen, NJ). Nonesterified fatty acids were determined using a diagnostic reagent kit (Wako Chemical USA, Richmond, VA) and triglycerides were determined using a trinder kit (Sigma Diagnostics, St Louis, MO).

### Intraperitoneal glucose tolerance test

10 to 16 week old male mice were injected intraperitoneally with 2.0 mg glucose per gram of body weight using a 20% (w/v) glucose solution (in 0.9% NaCl solution). Blood samples were collected from the hind limb saphenous vein at −15, 0, 15, 30, 60 and 120 min after injection and glucose levels were immediately measured.

### Real-time QRT-PCR

One step real time PCR was performed using the Mx3000P and Brilliant QRT-PCR reagents (Stratagene, Cedar Creek, TX). Total RNA was extracted from frozen mouse livers (14 to 20 week old male mice) using Trizol reagent (LifeTechnologies, Inc.) according to the manufacturer's protocol and stored at −80°C in 0.1 X SET (0.1% SDS, .5 mΜ EDTA, 1 mΜ Tris, pH 7.6). To prepare RNA for qRT-PCR, samples were precipitated in 100% ethanol, washed twice with 70% ethanol, then resuspended in autoclaved double-distilled water. Relative levels of PGC-1α, PEPCK, GLUT2, GCK, GAPDH, and G6Pase were determined using TaqMan probe and primer sets designed for each gene, and relative amounts of mRNA were determined based on a standard curve using Stratagene Mx3000P software.

### Statistical analyses

All data are presented as the mean ± standard error of the mean. Statistical significance was determined by one-way ANOVA and repeated measures ANOVA was used on the glucose tolerance tests followed by Tukey's Multiple Comparison Test. A *p-*value of less than 0.05 was considered statistically significant. Prism™ was used to plot data and determine statistical significance.
